# Enamel fluorosis related with fluoride-containing water ingestion and urinary excretion in schoolchildren 

**DOI:** 10.4317/jced.61052

**Published:** 2024-01-01

**Authors:** Farith González-Martínez, Ruth Gómez-Scarpetta, Olga-Bibiana Salcedo, Patricia Bermúdez-Reyes, Patricia Castro-Villamizar, María del Pilar Cerezo, Carmen Martínez, Lesbia Tirado-Amador, Andrés Salas-Zambrano, Alexandra Saldarriaga, Marisol Sánchez-Molina, Luis-Armando Vila

**Affiliations:** 1 DDS, MSPH, PhD. Public Health Research Group Universidad de Cartagena-Cartagena de Indias, Colombia; 2 Asociación Colombiana de Facultades de Odontología-Bogotá-ACFO, Colombia; 3 Latin American Network of Research on Fluorides and Dental Fluorosis, Cartagena 130014, Colombia; 4 BAM, MSc. Universidad Cooperativa de Colombia-Villavicencio, Colombia; 5 DDS, MSc. Universidad de Antioquia-Medellín, Colombia; 6 DDS, MSc. Universidad Cooperativa de Colombia-Envigado, Colombia; 7 DDS, MSc. Corporación Universitaria Rafael Núñez, - Cartagena, Colombia; 8 DDS, MSc. Universidad Autónoma de Manizales-Manizales, Colombia; 9 DDS, MSc. Universidad Santo Tomás-Bucaramanga, Colombia; 10 DDS, MSc. Universidad del Sinú-Cartagena, Colombia; 11 DDS, MSc. Universidad Cooperativa de Colombia-Pasto, Colombia; 12 DDS, MSc, PhD. Universidad CES-Medellín, Colombia; 13 DDS, MSc, PhDc Universidad Metropolitana-Barranquilla, Colombia; 14 DDS, MSc, PhD. Universidad del Magdalena-Santa Marta, Colombia

## Abstract

**Background:**

Natural water sources are considered as the major environmental exposure of fluoride, resulting in increased prevalence of enamel fluorosis. This type of natural exposure should be permanently monitored to avoid the interactions with other non-natural fluoride sources. We evaluated the prevalence of enamel fluorosis in Colombian schoolchildren and its relationship with fluoride-containing water ingestion exposure dose and urinary fluoride excretion.

**Material and Methods:**

We included 923 schoolchildren aged 7–12 years residing in eight municipalities in Colombia. Sampling of consumption water was performed in major aquifers used for daily supply. Samples were collected in 98 polyethylene containers and refrigerated until analysis. Water and urine fluoride concentrations were measured using the fluoride selective electrode method. Enamel fluorosis was evaluated using Thylstrup and Ferjerskov Index (TFI). Demographic and anthropometric characteristics were assessed. Besides, other exposures to non-natural fluoride were also evaluated. Logistic regression was applied for multiple analyses.

**Results:**

The median fluoride concentration in water and urine samples was 10.5 mg/L and 0.63 mg/L respectively, with the highest value found in Algarrobo-Magdalena, and the lowest value found in Manzanares-Caldas. The overall prevalence of enamel fluorosis was 86.1%, being more frequent the mild codes with TFI-1 to TFI-2. The highest prevalence was found in Margarita-Bolívar and Manzanares-Caldas, and the most severe codes (TFI-5 to TFI-9) were detected in Manzanares-Caldas. The multiple analysis revealed water ingestion exposure dose, urinary excretion, involuntary intake of toothpaste, amount of table salt consumption and sex as significant factors (*p*< 0.001).

**Conclusions:**

The fluoride ingestion exposure dose and its subsequent urinary excretion could be used as estimators of past fluoride exposure, explaining the current prevalence of enamel fluorosis in Colombian schoolchildren.

** Key words:**Fluoride, groundwater ingestion, enamel fluorosis, prevalence, severity.

## Introduction

Fluorine is a naturally occurring element with a widespread distribution, primarily in geological environments. It is also present in non-geological environments in substrates such as water, soil, air and vegetation (1). The most commonly identified compounds of environmental origin with fluorine content are fluorite (calcium fluoride), cryolite (sodium and aluminium fluoride), apatite (calcium phosphate) and phosphate rocks (2,3).

Artificial addition of fluorides to sources of intake such as water and Table salt has been performed globally since 1950 as the major strategy for controlling the progression of dental caries. Although drinking water in Colombia does not contain added fluoride since 1989, when the public policy was changed by adding fluoride to table salt, the country established a control and surveillance system for the quality of water for human consumption (SIVICAP), which guarantees the monitoring of fluoride concentration levels in drinking water, with the maximum acceptable value of fluorides in water being 1 mg/L (4,5). However, this system does not have sufficient coverage to monitor all sources of water access for the Colombian population, because several communities residing in rural areas have a service coverage of 74 % (6), and this consumption is supplied by unconventional aquifers that are generally not included in the water samplings performed by territorial entities.

Since 2012, the National Institute of Health of Colombia has implemented a sentinel surveillance programme for fluoride exposure (SIVIGILA), which monitors fluoride levels in drinking water and Table salt in some municipalities selected by the territorial entities. Between 2012 and 2015, the programme has identified 19 municipalities with fluoride values in water samples of >1mg/L. Similarly, during this same period, 53 municipalities were identified with fluoride values in table salt samples of >220 mg F/kg/salt (7). Although the programme is still active, it has a limitation of under-records in the data due to lack of samplings that represent the water and salt consumption of all participating populations.

Excessive intake of fluoride on a regular basis during the tooth formation period causes a clinical defect in enamel development, which is characterised by greater discoloration and porosity than the normal enamel and is known as enamel fluorosis (8,9). Although the optimal levels of daily fluoride intake are inconsistent, some studies indicate that intake doses of >0.07 mg F/kg w/day can cause dental fluorosis (10).

The last National Oral Health Survey performed in Colombia in 2014 (11), reported a prevalence of enamel fluorosis of 59.1%, confirming a significant increase compared with the National Oral Health Survey performed in 1998 (12), which reported a prevalence of 11.8 %. These data indicate possible failures in the implementation of public policy to control fluoride intake from different sources. It can be clearly recognised that massive access to fluoride sources has generated an effect on dental caries in the country by reducing its prevalence (11). Nevertheless, the non-beneficial effects of fluoride have increased concern at the level of government entities, to the point of being considered as a public health problem.

Chronic toxicity and the increase in the prevalence and severity of enamel fluorosis have been associated with exposure to different sources of fluoride administration, especially sources of systemic intake such as water, Table salt, toothpastes, fluoride supplements and diet (13-18). Therefore, the impact on chronic toxicity of the various sources of fluorides is related to several variables, including the time of exposure, concentration and quantity and frequency of consumption, as well as the urinary fluoride excretion, which become a highly complex exposure, making it difficult to control (19-21).

The above-described background information indicates the need to explore the effects of exposure to fluorides by groundwater ingestion, including in the explanatory models all the variables that intervene in this process. Therefore, we investigated the prevalence of enamel fluorosis in Colombian schoolchildren and its relationship with fluoride-containing water ingestion exposure doses and fluoride urinary excretion.

## Material and Methods

-Study population

This study was conducted on 923 schoolchildren aged 7–12 years residing in the following eight municipalities in Colombia with exposure to fluoride due to groundwater ingestion: Algarrobo-Magdalena, Campo de la Cruz-Atlántico, La Estrella-Antioquia, Manzanares-Caldas, Margarita-Bolívar, Nariño-Nariño, Oiba-Santander and Puerto López-Meta. Eligible participants enrolled in this study have been living in these municipalities since birth and consumed groundwater on a regular basis. Exclusion criteria were medical history of renal disease and severe malnutrition.

The sample size was determined based on a type I error of 5% and a power of 80%. Based on previous data, using the Stata® software (V12, Stata Corp. LP, College Station, TX, USA), the lowest expected frequency for the prevalence of dental fluorosis was calculated (11), resulting in a target sample size of 987 subjects. To account for possible incomplete processing of the questionnaire, the sample size was increased by 10 % (1086). Thus, a total of 1086 schoolchildren were recruited, and 163 were excluded because they did not fulfil the parameters cited earlier. The final study population consisted of 923 participants.

-Groundwater collection

**Figure 1:**
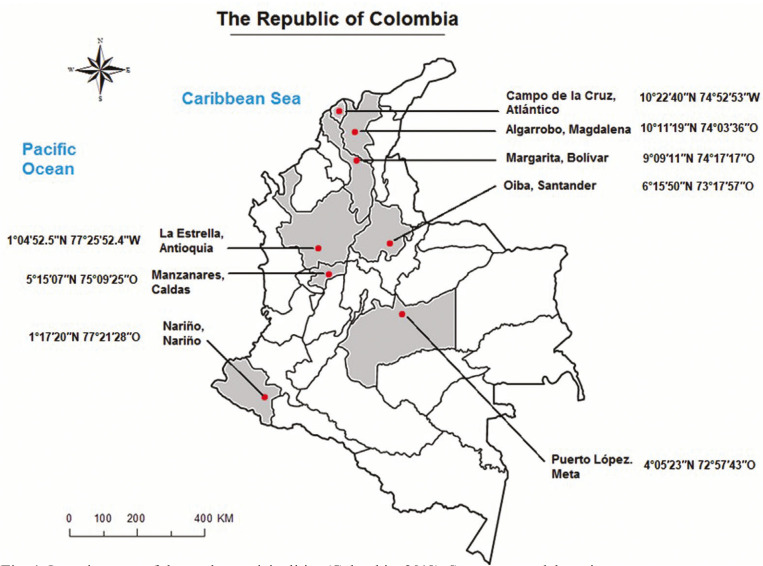
Location map of the study municipalities (Colombia, 2018). Source: own elaboration.

A total of 98 groundwater well sites were selected in the study municipalities (Fig. 1). Water samples were collected in 250-mL polyethylene bottles that were previously treated with 10 % HNO3 for 2 h, rinsed with ultra-pure water (18 MΩ•cm; distilled and deionised water) and dried (to avoid contamination). The containers were also rinsed three times with the same sampling water before sample collection. After sample collection, the containers were refrigerated (three Celsius degrees), transported and stored in the laboratory until analysis, (Fig. 1).

-Urine collection

The urine samples of the participants were collected in 50 mL sterile falcon tubes, taking a standard volume of 20 mL for analysis. This sample represents a single point in time, corresponding to the first morning urine. After sample collection, the containers were refrigerated (three Celsius degrees), transported and stored in the laboratory until analysis.

Questionnaire

A survey was performed to evaluate the demographic and anthropometric information of the study participants. Moreover, details regarding the ingestion of groundwater and other fluoride exposure sources such as consumption of packaged beverages, involuntary intake of toothpaste, amount of toothpaste used, frequency of teeth brushing and amount of table salt consumption were collected. These data were obtained through a self-administered questionnaire, with a supplementary interview. This questionnaire was evaluated before its application by three expert judges to confirm its appearance validity. Furthermore, a reliability analysis stability, test-retest was applied in a pilot group of volunteer parents at two time intervals to correlate the obtained two data distributions. In addition, all interviewers were trained to administer the assigned questionnaires. The questionnaires were also monitored during the data collection process through a random review to assess adherence to the protocols by the interviewers.

-Quantification of fluoride in groundwater and urine

Fluoride concentrations were measured using a potentiometer with fluoride ion-selective electrode (Thermo Scientific™ Orion™; Fisher cat. #13-642-285 Mfr. # 9609BNWP) (22,23). The water and urine samples were analyzed by duplicate adding TISAB II buffer with CDTA just before analysis and using standard reference material (NIST-USA, 1984: SRM 3183). The measurement procedures were performed at the Public Health Research Laboratory of the School of Dentistry, University of Cartagena, Colombia. Urinary fluoride concentrations were adjusted by dilution to eliminate variation from fluid balance. Therefore, urinary creatinine was determined by spectrophotometer Genesys 10S UV-VIS (Thermo Scientific, USA) and urinary fluoride concentrations were expressed as mg F per g creatinine.

-Determination of water ingestion exposure doses (IDag) 

Human exposure to fluoride in groundwater was evaluated using IDag (24). This index was expressed as risk using the reference dose (<0.05 mg/kg/day) and was calculated according to the following equation: IDag = (C × IR × EF) / (BW), where C is the fluoride concentration in water (mg/L), IR is the water intake rate per day (L/day), EF is the exposure frequency (days/year) (a value of 1 was used, representing daily exposure to fluoride) and BW is the body weight (kg) (the average body weight was determined as 32 kg).

-Clinical examination

The clinical signs of enamel fluorosis were evaluated using the TF index. These results are indicators of chronic fluoride exposure, and each indicator of clinical injury has been correlated with histological characterisation. This index classifies the severity of enamel fluorosis lesions among nine indicators as follows: (TFI-0) = absence of visible lesion and (TFI-1 to TFI-9) = presence of clinically visible lesions. Based on the severity categories, the indicators were classified as (TFI-1 to TFI-2) = mild fluorosis, (TFI-3 to TFI-4) = moderate fluorosis and (TFI-5 to TFI-9) = severe fluorosis (25). Two examiners received training for enamel fluorosis measurements using the TFI. Clinical calibration values included inter-examiner and intra-examiner comparison, using an external examiner as a reference point. The two examiners obtained agreement scores above 0.75 for the weighted kappa coefficient.

-Statistical analyses

Statistical analyses were performed using the R software (26). Proportions, absolute and relative frequencies and mean and standard deviation were used for statistical description. In addition, correlation coefficients were used to evaluate the relationship among the fluoride-containing water ingestion exposure dose and quantification of fluoride in urine. The prevalence of enamel fluorosis was calculated as the proportion of individuals with a given category by dividing into the total number of participants. The study population was divided into two subgroups for the purpose of analysis, and pairwise comparisons were performed between the presence and absence of enamel fluorosis and the severity levels according to TFI using the chi-square (χ²) test (*p* < 0.05). A complementary analysis was performed to understand the significance of differences between the presence and absence of enamel fluorosis according to the low and high exposure dose, effect size test (27). A multivariate logistic regression analysis was performed incorporating enamel fluorosis prevalence as a dependent variable and water ingestion exposure dose, urine excretion, involuntary intake of toothpaste, amount of Table salt as independent variables. Potential confounders (age, sex, body mass index (BMI) and exposures to fluoride other than groundwater ingestion) were also considered in this study. Interaction terms among the water ingestion exposure dose that provided a *p* <0.2 and potential confounders were evaluated.

## Results

-Characterisation of the study municipalities

This study was conducted in eight municipalities in Colombia, distributed by different geographical areas, especially rural areas. The latitude and longitude of the study locations are depicted in Figure 1.

-Characteristics of study subjects

The mean age, height, weight and BMI of the study population were 9.5 ± 0.04 years, 1.4 ± 0.003 m, 31.9 ± 0.23 kg and 18.7 kg/m2, respectively. Girls comprised 50.2 % of the study population, and the most frequent (20.8%) school grade was fourth grade of elementary school. An amount of Table salt consumption of ≥1 spoonful (75.4 %) and an amount of toothpaste of more than or equal to half of the toothbrush (54.8 %) showed the highest frequency among fluoride exposure factors. The other characteristics are shown in Table 1.

**Table 1:**
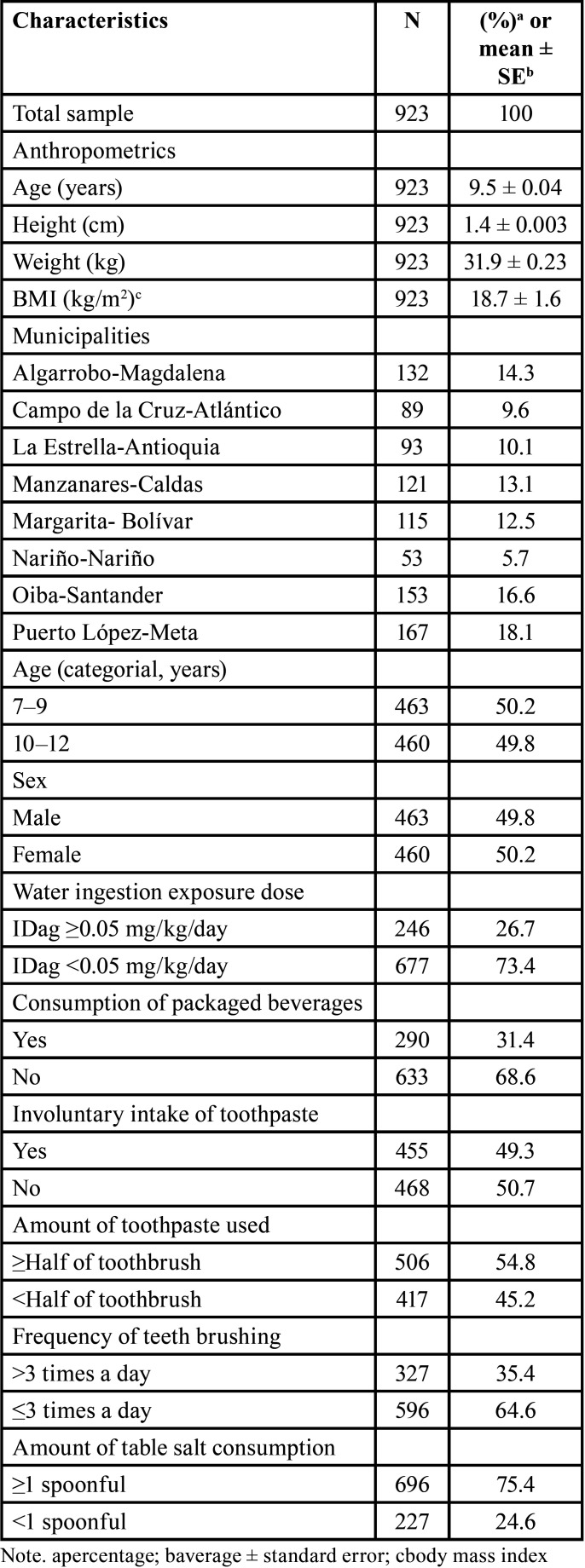
Characteristics of the study population.

-Concentration of fluoride in drinking water

The water samples contained a fluoride concentration of 0.02 to 24.3 mg/L, with the overall mean concentration being 4.32 ± 8.35 mg/L. Algarrobo-Magdalena (24.3 ± 26 mg/L) and Margarita-Bolívar (6.3 ± 5.7 mg/L) were the municipalities with the highest concentration of fluoride in drinking water (Table 2).

**Table 2:**
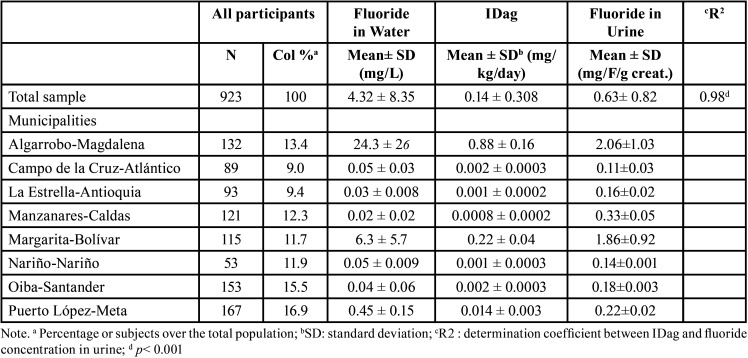
Fluoride water ingestion exposure dose (IDag) and fluoride concentration in water and urine by municipalities.

-Water ingestion exposure dose (IDag) and Fluoride concentration in urine

The IDag values ranged from 0.0008 to 0.88 mg/kg/day, and the median value was 0.002 mg/kg/day (0.001–0.17 mg/kg/day). The highest IDag values were found in Algarrobo-Magdalena (0.88 mg/kg/day) and Margarita-Bolívar (0.22 mg/kg/day), above reference dose (<0.05 mg/kg/day). The urine samples contained a range fluoride concentration of 0.11-2.06 mg/F/g-creatinine with the overall mean concentration being 0.63 ± 0.82 mg/F/g-creatinine. The individuals residing in the municipalities of Algarrobo-Magdalena and Margarita-Bolívar showed the highest concentrations with 2.06 and 1.86 mg/F/g-creatinine respectively. Besides, the mean fluoride-containing water ingestion exposure dose (IDag) and the mean fluoride concentration in urine showed a positive and statistically significant correlation (r = 0.98; *p* < 0.001), (Table 2).

-Prevalence and severity of enamel fluorosis in schoolchildren

The overall prevalence of fluorosis among the 923 children was 86.1 %. The highest dental fluorosis prevalence was found in Margarita-Bolívar and Manzanares-Caldas. We also detected a significant association between enamel fluorosis prevalence and municipalities (*p* < 0.001) and between enamel fluorosis prevalence and sex (*p* = 0.03) (Table 3).

**Table 3:**
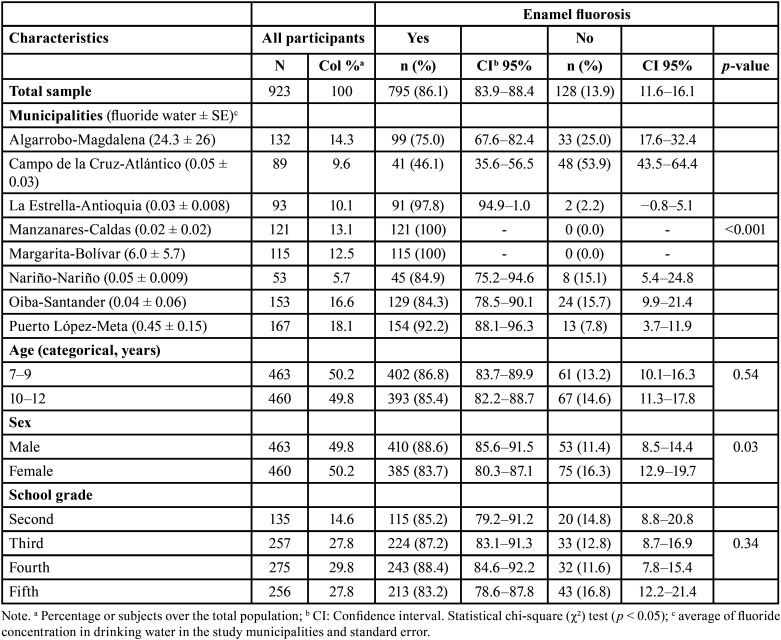
Enamel fluorosis prevalence in schoolchildren by characteristics of the study population.

Regarding severity, the most frequent was mild fluorosis (42.5 %), followed by moderate fluorosis (33.6%). The most severity codes were found in Manzanares-Caldas (TFI-5 to TFI-9; 39.7%). We also found an association between TFI and municipalities (*p* < 0.001), between TFI and sex (*p* = 0.03) and between TFI and age (*p* = 0.009) (Table 4).

**Table 4:**
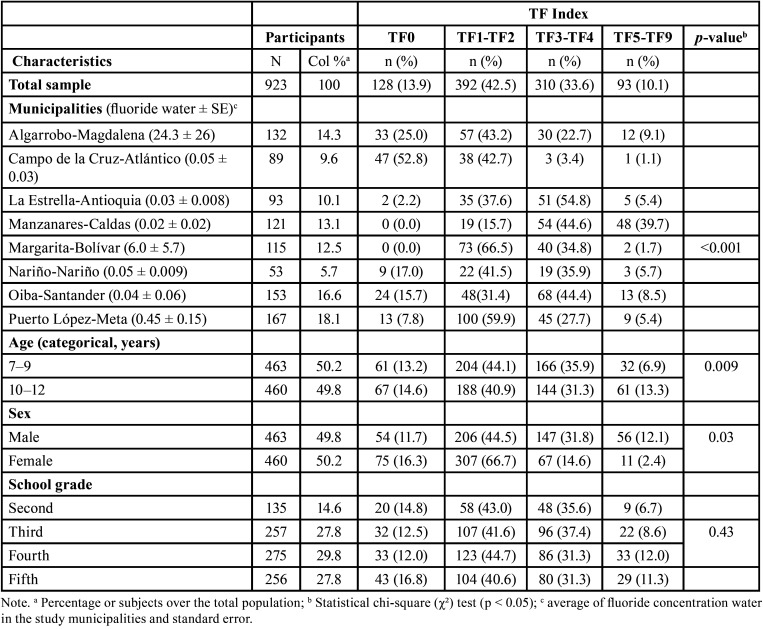
Enamel fluorosis severity (TFI) in schoolchildren by characteristics of the study population.

-Factors associated with enamel fluorosis prevalence

The risk factors associated with enamel fluorosis were involuntary intake of toothpaste (OR = 1.96 ± 0.39; *p* < 0.001) and amount of Table salt consumption (OR = 3.58 ± 0.71; *p* < 0.001). The other factors were not statistically significant (Table 5).

**Table 5:**
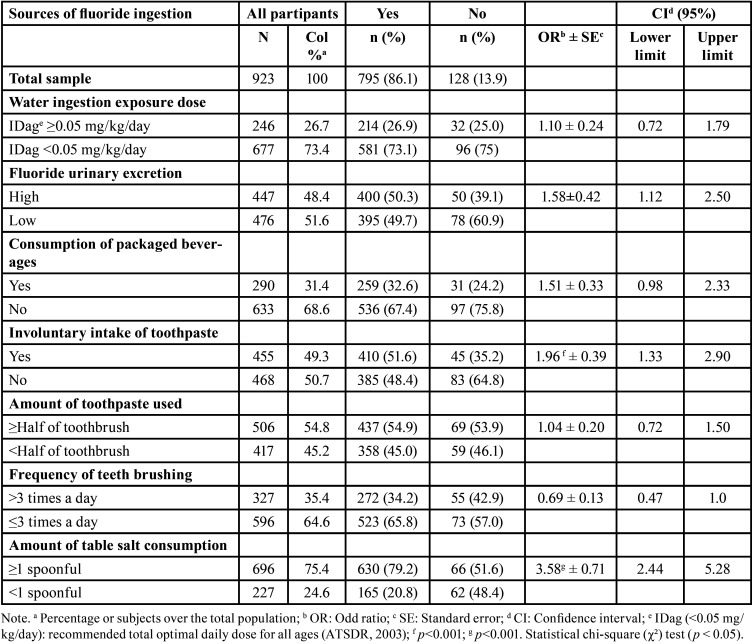
Fluoride exposure related with enamel fluorosis prevalence in schoolchildren.

-Multiple analyses between exposure factors and enamel fluorosis prevalence

Binomial analyses were conducted to determine the best explanatory model of enamel fluorosis. The outcome showed a statistically significant likelihood ratio (*p* < 0.001). The raw and adjusted models were compared. It was observed an increase in the statistical strength of the association by the effect of the interactions between the following exposure factors; water ingestion exposure dose, urinary excretion, involuntary intake of toothpaste, amount of Table salt and sex, being the coefficient of determination slightly higher (R2 = 30%) (Table 6).

**Table 6:**
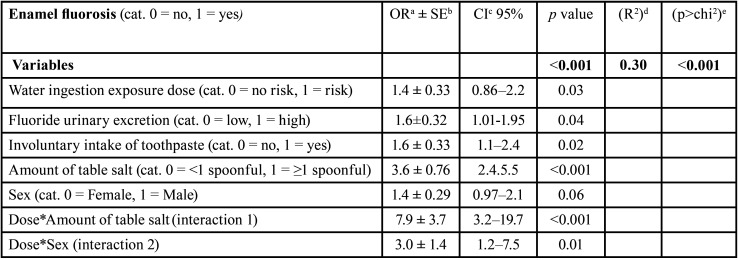
Binomial model between exposure to fluoride and enamel fluorosis prevalence, adjusted by covariates, in schoolchildren.

## Discussion

In Colombia, fluoride is added to Table salt as a measure of public health welfare for the prevention of tooth decay (28). However, fluoride can also occur naturally in geological environments (2,29). Therefore, basic instruments have been established for the control and surveillance of the quality of water for human consumption, because unconventional water sources used for human consumption can be considered to have high risk for the presence of enamel fluorosis. The participants of the present study consumed water from underground sources, due to which it is necessary to understand the exposure in detail.

The ingestion of fluoride within the ‘optimal range’ (0.05–0.07 mg F/kg/day) (30), provides concentrations required in the oral environment to inhibit mineral loss or promote mineralisation in the tooth enamel (31). In the present study, the results showed that the fluoride ingestion level through drinking water was below the ‘optimal range’. However, in two municipalities (Algarrobo-Magdalena and Margarita-Bolívar), the fluoride ingestion levels could be considered as a risk factor for enamel fluorosis, which are possible fluorosis-endemic areas. Whereby, the fluoride concentration by ingestion of water is an indicator reflecting ingestion of water source from environmental fluoride. As most fluoride ingestion in the municipalities of Colombia was from groundwater, it is necessary to monitor fluoride levels in drinking water in different areas and over time.

Several indicators have been reported to calculate the water ingestion exposure doses (24), although is clear that the fluoride concentration in water has a high impact within the equation used to calculate the ingestion exposure dose to fluoride, other factors such as the frequency of water ingestion and body weight also contribute. The World Health Organisation recommended drinking water fluoride levels of 0.5–1.5 mg/L for the prevention of tooth decay (32). Furthermore, in countries as Canada and E.U., the recommended concentration is 0.7 mg/L (33,31). In our study, of the 94 selected water samples, 25.5% had fluoride concentrations greater than the recommended values for Colombia (1 mg/L) (5). Therefore, the schoolchildren with the highest exposure in terms of fluoride concentration and frequency of ingestion of water showed the highest water fluoride ingestion dose. This suggests the need to monitor environmental variables for the epidemiological surveillance of fluoride exposure.

When we examined the association between exposure to fluoride in water and the prevalence of enamel fluorosis, the results were not consistent because of the higher prevalence of enamel fluorosis in the municipality Manzanares-Caldas, although it showed the optimal concentrations of fluoride in water, whereas in the municipality Algarrobo-Magdalena with the highest concentrations of fluoride in water, the prevalence and severity of enamel fluorosis were lower. These findings could be explained by the environmental (temperature) and geographical (altitude) differences between the study municipalities. According to the altitude, a greater prevalence of enamel fluorosis has been reported in populations residing in high-altitude cities (10), due to hypoxia, which decreases urinary pH and increases the retention of fluoride. Similarly, populations living in cities with lower temperatures have reduced energy demand, causing the excretion of fluoride due to an increase in the retention rate (34). This finding suggests a plausible conjecture, because Manzanares-Caldas has an average temperature of 19°C and an altitude of 1933 m above sea level, whereas Algarrobo-Magdalena has an average temperature of 27.8°C and an altitude of 24 m above sea level. However, this hypothesis must be tested in further studies.

Based on the presence of enamel fluorosis, the present study showed a higher prevalence and severity than the national data for Colombia (11). These high data may be explained by the different sources of fluoride exposure that contribute to increasing the daily ingestion dose (water and Table salt, toothpastes, mouthwash and food and drinks, as well as application of dental fluoride) (35-41). Similarly, the presence of other factors can increase the individual susceptibility to the toxic effects of fluoride, such as altitude and environmental temperature, malnutrition, diet components, gastric and urinary pH, kidney failure and genetic predisposition (10,42,43,20).

In the bivariate analyses, we found no association between the fluoride-containing water ingestion dose and prevalence of enamel fluorosis, but there was an association between the amount of Table salt consumption and involuntary intake of toothpaste. Although a surveillance and control system was implemented in Colombia to monitor fluoride levels in table salt for compliance with optimal levels (180–220 mg/F kg/salt) (7), the issue of the amount of table salt consumed daily is evident. The global average salt consumption in the country is 10–15 g/day (44), although the recommended amount is 5 g/person/day, which is sufficient to maintain adequate concentrations of fluoride in the saliva for the prevention of tooth decay (45,46). Similarly, the National Nutritional Status Survey in Colombia reported a high amount of Table salt consumption (47). The majority of participants in our study responded affirmatively to the preparation of food with the amount of table salt higher than the recommended level, which is an important risk factor when several sources of excessive fluoride intake are combined.

The involuntary intake of toothpaste during tooth brushing is a common habit in children aged <5 years in Colombia (11). In our study, a high percentage of parents did not monitor the amount of toothpaste used by children, and moreover, children aged between 2 and 4 years used adult toothpaste with fluoride concentrations above the recommended level (1450 mg/L). These data demonstrate the inappropriate use of toothpaste with high fluoride concentrations among children. However, according to risk, some studies have reported that the use of toothpastes with high fluoride content did not increase the urinary fluoride excretion significantly (48,49). Similarly, these habits may contribute together with the fluoride content in water and Table salt to increase the cumulative dose above the optimal levels (50,17). Several authors reported that the involuntary intake of toothpaste in 5-year-old children contributes 30% of the total fluoride ingestion, which is higher than the same contribution in adults (51,52). In the present study, the data for the involuntary intake of toothpaste were obtained from questionnaires that reconstructed the past exposure. It is clear that this method is not highly sensitive to evaluate this exposure; therefore, we recommend conducting longitudinal studies on urinary fluoride excretion to confirm the weight of this risk factor on the presence of enamel fluorosis.

In this regard, it is evident that the impact on chronic toxicity of the different fluoride sources, independent or in combination, is dependent on the time of exposure, concentration of sources and the amount and frequency of its consumption. Therefore, we conducted a multiple analysis to estimate the impact of the effect of the combination of various fluoride sources and demographic variables and obtained a multiple association model with statistical significance, where we tested the interactions between the dose of water intake, involuntary toothpaste intake, amount of table salt intake, fluoride urinary excretion and sex of the participants. We found that the variable sex influenced the model from the category of boy or girl, indicating that boys probably ingested greater amounts of water or Table salt than girls, due to greater muscle mass that consequently increases the intake needs (53). Likewise, the strength of association of urinary fluoride excretion was increased in multiple analysis, probably due to the strong positive correlation with the water ingestion exposure dose, showing a higher urinary fluoride concentration in children with high levels of water ingestion fluoride exposure dose. These outcomes have also been reported by other studies, although with great variability (29,54-57).

Among the possible limitations inherent to the type of this study (cross-sectional design) are the evaluation of enamel fluorosis and exposure to fluoride at the same time point, reconstructing the exposure in the stage where it originated (between 1 and 4 years of age) and the possible memory bias that could incur in the parents’ responses when trying to reconstruct the past exposure using a validated questionnaire. It is important to emphasise that only children born and living in the respective municipalities were included in the present study, due to which it was expected that the fluoride concentrations currently found in natural water sources would be similar to those that had been exposed in the past, considering that none of the municipalities reported substantial improvements in the water source in the past 15 years.

The findings of this study provide relevant information for public policies on oral health in Colombia, focusing on the deficiencies in the current fluoride exposure control and surveillance system, which monitors and controls only two of the various sources of fluoride involved (natural water and table salt). Which shows a low efficacy of the government strategy used in Colombia to reduce the chronic toxic effects of fluoride on the dental enamel of people.

To conclude, the prevalence of enamel fluorosis in Colombian schoolchildren can be explained by some factors related to the fluoride-containing water exposure dose, other sources of exposure and some demographic factors.
